# Increased survival reveals new paradigms in diabetic macular edema among patients with type 1 diabetes

**DOI:** 10.1007/s12020-025-04295-8

**Published:** 2025-06-02

**Authors:** Pedro Marques-Couto, Arthur Villard, Pedro Mota-Moreira, António Ferrão-Mendes, Ana Rita Leite, João Sérgio Neves, Manuel Falcão, Rita Laiginhas

**Affiliations:** 1 Department of Ophthalmology, Unidade Local de Saúde de São João, Porto, Portugal; 2 Faculty of Medicine of the University of Porto, Porto, Portugal; 3 Department of Endocrinology, Diabetes and Metabolism, Unidade Local de Saúde de São João, Porto, Portugal; 4 Department of Surgery and Physiology, Faculty of Medicine of the University of Porto, Porto, Portugal

**Keywords:** Type 1 diabetes, Diabetic macular edema, Diabetic retinopathy, Risk factors, Longitudinal study

## Abstract

**Purpose:**

Treatment and monitoring developments markedly improved the survival rate of individuals with type 1 diabetes (T1D). Nevertheless, literature remains scarce on long-term incidence of diabetic macular edema (DME) in this population, with the major study existing in the field – Diabetes Control and Complications Trial/Epidemiology of Diabetes Interventions and Complications (DCCT/EDIC) – reporting its development at 30 years of disease. We aimed to estimate the long-term risk of DME and associated factors in a representative sample of type 1 diabetic patients.

**Methods:**

This retrospective cohort study included 235 patients with T1D from a tertiary center in Portugal. Data on demographics and clinical characteristics were extracted from digital medical records and analyzed using descriptive statistics. Kaplan-Meier survival analysis was used to assess DME development over the follow-up period. Potential predictors of DME, including age at T1D diagnosis, duration of T1D, glycated hemoglobin (HbA1c), body mass index (BMI), severity of diabetic retinopathy (DR), systolic and diastolic blood pressure (SBP and DBP), and smoking habits, were analyzed using multivariate Cox regression.

**Results:**

The overall prevalence of DME was 18.7%. The cumulative risk of developing DME escalated from 7.6% at 20 years to 51.1% at 65 years of T1D duration (*p* < 0.001). Patients with severe or proliferative DR had a significantly higher prevalence of DME (*p* < 0.001). The prevalence of microvascular complications was higher in the DME group (54.5% versus 19.4%, *p* < 0.001). Age at T1D diagnosis and DR severity were the strongest predictors of DME, with hazard ratios of 1.03 (95% CI [1.00–1.06]; *p* = 0.029) and 1.46 (95% CI [1.20–1.77]; *p* < 0.001), respectively. HbA1c and SBP were associated with DME in univariate analysis but lost significance in multivariate models.

**Conclusions:**

This study highlights the time-dependent nature of DME development in T1D, with a marked increase in risk beyond 20 years of disease duration, a pattern that appears to differ from what is typically observed in T2D. DR severity was a key predictor of DME. DME was associated with the presence of microvascular, but not macrovascular complications. These findings emphasize the importance of tailored surveillance strategies to improve outcomes in this high-risk population.

## Introduction

Advancements in understanding β-cell activity, glucose physiology and immunological pathways have led to innovative treatments and developments in the management of type 1 diabetes (T1D). Insulins with advanced pharmacokinetic and pharmacodynamic profiles, new methods to monitor blood glucose, and rigorous management of cardiovascular risk factors are increasing the survival rate for these patients [1]. A recent study covering 201 countries reported that, in 2021, there were 8.4 million people worldwide with T1D, with 1.6 million (19%) aged 60 years or older. This study also highlighted that a 10-year-old diagnosed with T1D in high-income countries could expect a remaining life expectancy of up to 65 years [2].

Diabetes can lead to target organ damage, with diabetic retinopathy (DR) being one of the primary complications [3]. Diabetic macular edema (DME) is a sight threatening complication of DR [4–6], which consists in the accumulation of intraretinal fluid in the macular region due to a disruption of the blood-retinal barrier. Hyperglycemia-related endothelial cell damage increases vascular permeability, causing an imbalance between fluid entry and exit processes [7, 8].

DME is a major etiology of visual impairment in type 2 diabetes (T2D). In T1D, however, proliferative diabetic retinopathy (PDR) is the most common vision-threatening complication and DME-related visual impairment has been shown to be less common [9]. The Wisconsin Epidemiologic Study of Diabetic Retinopathy (WESDR) initially demonstrated a strong association between visual impairment in patients with T1D and the severity of retinopathy plus DME at baseline, with a 25-year cumulative incidence of DME of 29%. This study suggested that better glycemic and blood pressure control at baseline and throughout the study were beneficial in reducing the incidence of DME, which was expected to occur with time. Despite its importance, the study population consisted of a sample selected from patient with diabetes who received primary care from 1979–1980 [5], which is not representative of the current era of new trends in metabolic control and treatments. A more recent study evaluated the thirty-year time trends in DME in youth with T1D up to 2019 concluding that the overall prevalence of DME was 0.5–1.4%, and other studies with similar results corroborated the hypothesis that the improvements in both diagnostic accuracy and treatment possibilities of DME during the recent years have played an important role in this phenomenon [10, 11]. However, due to the crucial role of diabetes duration in the development of microvascular complications in T1D, prevalence may not properly reflect the risk of DME in this population, and time-dependent analysis is more likely to represent the real trends. Revisiting the natural history of DME in T1D is particularly important as survival rates for patients with T1D increase. Longer disease durations were not adequately represented in existing studies, and since DME poses a significant threat to vision, its early detection is crucial, and current screening strategies may need adjustments.

The aim of the present study is to evaluate the natural history of DME in patients with T1D, current epidemiological trends, and potential predictors for its development, using a time-dependent analysis.

## Material and methods

### Study design

This observational, retrospective, cohort study including patients with T1D followed in a Portuguese tertiary care center (Unidade Local Saúde S. João – ULS São João, Porto, Portugal). All methods adhered to the ethical guidelines set by the Ethics Committee of Centro Hospitalar de São João/Faculty of Medicine of Porto University, as well as the principles outlined in the 1964 Helsinki Declaration and subsequent revisions.

### Sampling

The study target population consisted of patients aged 18 years or older diagnosed with T1D and covered by the ULS São João catchment area in 2017. As stated by the national epidemiology statistics from 2011, the reference area of this tertiary center has a population of approximately 340,000 people, of whom around 200,000 are aged 18 years or older [12]. According to a Portuguese study, the prevalence of T1D in the Northern Region of Portugal is estimated to be 1.66 cases per 1000 inhabitants [13]. Based on these estimations, a population of around 332 patients with T1D was expected to exist in this referral region. By merging medical records, 309 patients met the inclusion criteria. Exclusion criteria included incomplete ophthalmologic evaluation not allowing the determination of the presence of DME, patients who had an interval between consecutive evaluations of more than 3 years, doubtful diagnostic between T1D and T2D or maturity-onset diabetes of the young (MODY). After applying these criteria, a total of 235 patients were included. As the sample size aligned with the initial estimation, it was considered as a proper and representative sample of the target population. All available medical records and visits were retrospectively reviewed from the first available visit up to January 2024.

### Clinical and laboratorial records

Data regarding gender, age at the time of baseline examination, age at T1D diagnosis, duration of disease, mean hemoglobin A1c (HbA1c), body max index (BMI), smoking status, severity of DR, mean systolic blood pressure (SBP), mean diastolic blood pressure (DBP), presence of microvascular complications and DME were collected.

Disease duration was designated as the period between the age of T1D diagnosis and the last ophthalmological evaluation. This variable was categorized into 6 smaller groups (less than 5, 10, 15, 20, 25 and ≥25 years) and into 2 larger groups: <20 years and ≥20 years.

Mean HbA1c, mean SBP, mean DBP and mean BMI were defined as the average of all measurements found in our electronic medical records. Smoking status was defined as present/absent.

DR was classified as absent, mild non-proliferative retinopathy (NPDR), moderate NPDR, severe NPDR and PDR, according to a combination of clinical findings in fundoscopic evaluation and fluorescein angiography (FA). DR was defined as absent if there were no signs and evidence of any DR manifestation, such as intra-retinal hemorrhages, cotton wool spots or microaneurysms. Patients with panretinal photocoagulation were also included in the PDR group. If there was asymmetric retinopathy, the eye with the worst condition was used for classification. Microvascular complications were defined as the presence of nephropathy or neuropathy, as retrieved from medical records. Macrovascular complications were defined as the presence of peripheral artery disease, acute myocardial infarction and stroke, as retrieved from medical records. DME was classified as present/absent according to a combination of findings in the fundoscopic examination, Optical Coherence Tomography (OCT) scan and FA evaluation, as available.

### Statistical methods

Descriptive statistics were computed to summarize the demographic and clinical characteristics of the study population. Normal distribution was assessed by histogram visual inspection. Continuous variables are presented as mean ± standard deviation when the data followed a normal distribution, or as median and interquartile range (IQR) when the data followed a skewed distribution. Categorical variables were described as frequencies. The cumulative risk of developing DME over time was estimated by the Kaplan-Meier method. Survival analysis and logistic time-dependent regression analysis, using uni- and multivariate Cox regression models adjusted for disease duration, were performed to identify factors associated with the presence of DME. To analyze continuous variables with normal distribution, a T-test for independent samples was performed. Regarding continuous variables with a skewed distribution, a Mann-Whitney test was performed. Statistical significance was set at a p-value of less than 0.05. The statistical analysis was performed using IBM SPSS Statistics 27 software.

## Results

### Baseline characteristics of the sample

A total of 235 participants with T1D were included in the study, of whom 119 (51%) were male, and 63 (27%) had severe NPDR or PDR. The median age of the participants was 44 (21) years, and the median age at T1D diagnosis was 18.3 (16.7) years. The median duration of T1D was 24 (14) years. The median HbA1c was 8.1 (2) %, and the median BMI was 24.2 (4.9) kg/m^2^. The mean SBP was 130 ± 14 mmHg, while the mean DBP was 74 ± 9 mmHg. All baseline characteristics of the participants are summarized in Table 1.

**Table 1:** Clinical and biochemical characteristics of type 1 diabetes patients included in the study

	Total (*n* = 235)	No Macular Edema (*n* = 191)	Macular Edema (*n* = 44)	*p*
Sociodemographic Factors				
Sex (male), n (%)	119 (51%)	100 (52.4%)	19 (43.2%)	0.272
Age at last follow-up, median (IQR) (years)	44 (21)	44 (20)	46 (22)	0.611
T1D-related Factors				
Age at T1D diagnosis, median (IQR) (years)	18.3 (16.7)	18.2 (16.2)	20.3 (17.3)	0.633
T1D duration, median (IQR) (years)	24 (14)	23 (14)	25.5 (10)	0.341
Advanced DR, n (%)	63 (27%)	33 (17%)	30 (68%)	<0.001*
HbA1c, median (IQR) (%)	8.1 (2)	8.02 (1.8)	8.4 (1.8)	0.141
Decade of diagnosis, n (%)				
−1950’s	1 (0.4%)	1 (0.5%)	0 (0%)	
−1960’s	6 (2.6%)	4 (2.1%)	2 (4.5%)	
−1970’s	15 (6.4%)	8 (4.2%)	7 (15.9%)	
−1980’s	41 (17.4%)	25 (13.1%)	16 (36.4%)	
−1990’s	72 (30.6%)	56 (29.3%)	16 (36.4%)	
−2000’s	72 (30.6%)	69 (36.1%)	3 (6.8%)	
−2010’s	28 (11.9%)	28 (14.7%)	0 (0%)	
Co-morbidities				
BMI, median (IQR) (kg/m^2^)	24.2 (4.9)	24.3 (4.9)	24.02 (4.3)	0.781
Systolic Blood Pressure, mean ± SD (mm Hg)	130 ± 14	128 ± 13	137 ± 16	<0.001^*^
Diastolic Blood Pressure, mean ± SD (mm Hg)	74 ± 9	74 ± 9	72 ± 10	0.134

*BMI* body mass index, *DR* diabetic retinopathy, *HbA1c* glycated hemoglobin, *IQR* interquartile range, *SD* standard deviation, *T1D* type 1 diabetes mellitus

*p < 0.05

### Demographic and clinical characteristics of patients with DME

Over the years, 44 (18.7%) patients were diagnosed with DME, of whom 19 (43.2%) were male. Demographic and clinical characteristics of patients with and who did and did not develop DME are presented in Table 1. Patients with DME were older at the time of T1D diagnosis: 20.3 (17.3) years compared to 18.2 (16.2) years in those without DME, although no statistically significant difference was observed (*p* = 0.633).

Overall, patients that developed DME had a longer duration of T1D, with a median duration of 25.5 (10) years, compared to 23 (14) years in those without DME, although there was no statistically significant difference (*p* = 0.341). The median HbA1c levels were similar between the two groups: 8.4 (1.8) % in the DME group *versus* 8.02 (1.8) % in the non-DME group (*p* = 0.141). Additionally, the median BMI was comparable between the DME and non-DME groups (24.02 (4.3) kg/m² versus 24.3 (4.9) kg/m², respectively; *p* = 0.781).

The mean SBP was higher in patients with DME, with a mean of 137 ± 16 mmHg, compared to 128 ± 13 mmHg in those without DME (*p* < 0.001). However, DBP was similar between the two groups, with a mean of 72 ± 10 mmHg in the DME group and 74 ± 9 mmHg in the non-DME group (*p* = 0.134).

Patients with DME had a higher prevalence of microvascular complications compared to those without DME (54.5% *versus* 19.4%; *p* < 0.001). The prevalence of neuropathy was 38.6% in the DME group and 11.5% in the non-DME group (*p* < 0.001). Similarly, nephropathy was more frequent in the DME group compared to the non-DME group (43.2% *versus* 12.0%, *p* < 0.001). Regarding macrovascular complications, although the DME group showed a higher prevalence than the non-DME group (13.6% *versus* 6.3%), this difference was not statistically significant (*p* = 0.116).

Upon categorizing patients into two groups based on DR stage (minimal and moderate – non-advanced versus severe and proliferative - advanced), the prevalence of microvascular complications was higher in the advanced retinopathy group compared to the non-advanced retinopathy group (54% versus 15.7%, *p* < 0.001). Regarding neuropathy, its prevalence was also higher in the advanced group compared to the non-advanced group (36.5% vs 9.3%, *p* < 0.001). Similarly, nephropathy was more prevalent in the advanced group (42.9% vs 8.7%, *p* < 0.001). Macrovascular complications had a significantly higher prevalence in the advanced retinopathy group comparing with the non-advanced group (19% versus 3.5%, *p* < 0.001). Among patients with non-advanced retinopathy, there was a significant association between DME and neuropathy (*p* = 0.01), whereas no such association was found in the advanced retinopathy group (*p* = 0.283). Regarding nephropathy, a significant association with DME was observed in patients with advanced retinopathy (*p* = 0.035), but not in those with non-advanced retinopathy (*p* = 0.350).

Finally, concerning macrovascular complications, a significant association with DME was found in the non-advanced retinopathy group (*p* = 0.008), while no significant difference was observed in the advanced retinopathy group (*p* = 0.081).

### Duration of T1D

At first, the duration of T1D was stratified into 6 categories: less than 5, 10, 15, 20, 25 and ≥25 years. The prevalence of DME was 2.3, 0, 4.5, 15.9, 18.2 and 59.1%, respectively. Upon categorizing patients into two groups also based on T1D duration (<20 years and ≥20 years), a statistically significant difference was observed (22.7% *versus* 77.3%; *p* < 0.001).

### Severity of DR

Regarding patients with DME, the proportion of patients with mild NPDR, moderate NPDR, severe NPDR and PDR was 9.1, 22.7, 13.6 and 54.5%, respectively. Upon categorizing patients into two groups based on DR stage (minimal and moderate *versus* severe and proliferative), a statistically significant difference in the prevalence of DME was also demonstrated (31.8% *versus* 68.2%; *p* < 0.001).

### Survival analysis and potential predictors of DME

**Fig. 1:**
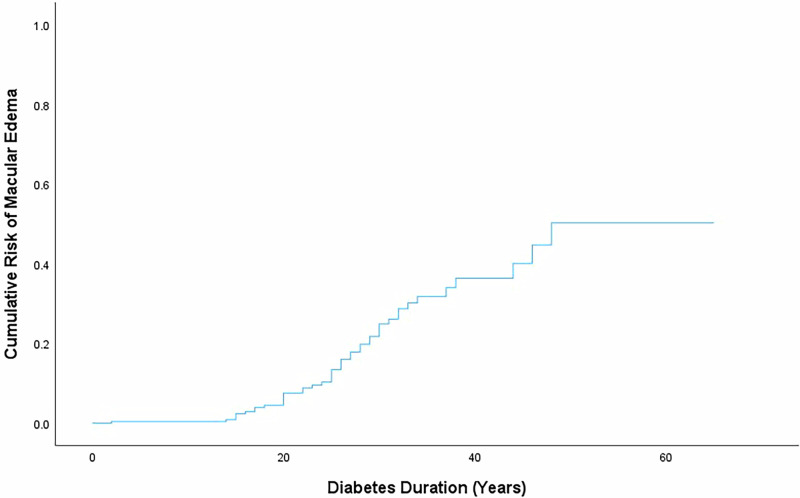
Kaplan-Meyer curve for risk of macular edema development

In a time-dependent survival analysis, the cumulative risk of developing DME escalated from 7.6% at 20 years of disease duration to 51.1% at 65 years of disease duration (Fig. 1). We performed a Cox-regression analysis to identify potential predictors for DME development, including gender, age at T1D diagnosis, decade of diagnosis, mean HbA1c, mean BMI, DR severity, smoking, mean SBP and mean DBP (Table 2). Among these factors, age at T1D diagnosis (HR: 1.04, 95% CI [1.01–1.06]; *p* = 0.015), mean HbA1c (HR: 1.26, 95% CI [1.11–1.57]; *p* = 0.037), DR severity (HR: 1.53, 95% CI [1.26–1.86]; *p* < 0.001) and mean SBP (HR: 1.03, 95% CI [1.01–1.05]; *p* = 0.012) showed a significant association with DME in univariate analysis. However, only age at T1D diagnosis (HR: 1.03, 95% CI [1.00–1.06]; *p* = 0.029) and DR severity (HR: 1.46, 95% CI [1.20–1.77]; *p* < 0.001) remained statistically significant after multivariate analysis, as depicted in Table 2.

**Table 2:** Univariate and final multivariate analysis of potential predictors of macular edema

	Univariate		Multivariate	
Variable	HR (95% CI)	*p*	HR (95% CI)	*p*
Sex	1.13 (0.62–2.05)	0.698		
Age at T1D diagnosis, years	1.04 (1.01–1.06)	0.015^*^	1.03 (1.00–1.06)	0.029^*^
Decade of diagnosis	0.11 (0.81–1.53)	0.507		
DR severity	1.53 (1.26–1.86)	<0.001^*^	1.46 (1.20–1.77)	<0.001*
Mean glycated hemoglobin, %	1.26 (1.11–1.57)	0.037^*^	—	0.390
BMI, kg/m^2^	1.01 (0.95–1.08)	0.706		
Smoking	1.11 (0.59–2.11)	0.746		
Mean systolic pressure, mmHg	1.03 (1.01–1.05)	0.012^*^	—	0.054
Mean diastolic pressure, mmHg	1.02 (0.98–1.05)	0.349		

*T1D* type 1 diabetes mellitus, *DR* diabetic retinopathy, *BMI* body mass index

*p < 0.05

## Discussion

This study offers significant insights into the current trends in cumulative risk and of DME in patients with T1D, emphasizing the roles of disease duration and severity of DR, as well as the crucial role of considering time-dependent analysis when evaluating these patients.

Existing literature suggests that the prevalence of DME in T1D is low. Although there are some studies addressing the prevalence of DME in patients with T1D, few have extended over a considerable follow-up time. A Brazilian cross-sectional study reported a DME prevalence of 1.3–7.1%, however, with shorter disease duration (15.5 ± 9.3 years) [14]. Another Brazilian cross-sectional found a prevalence of clinically significant macular edema (CSME) at 9.4%, with a mean disease duration of 14.4 ± 7.3 years, noting an increasing frequency of CSME with greater DR severity [15]. The research by Hainsworth et al. revealed that approximately 60% of patients remained CSME-free after 6.5 years of follow-up [16]. The higher prevalence in this latter study may be due to the lack of intensive therapy for most of these patients. Hietala et al., observed a CSME prevalence of 18%, with a similar mean disease duration to our cohort (24.6 ± 11.6 years) [17]. A Norwegian cross-sectional study, with a mean T1D duration of 19 years and a range similar to ours (up to 63 years), reported a 8% DME prevalence, likely explained by the shorter mean disease duration [18]. In Denmark, a study reported a prevalence of 7.9% with a lower mean diabetes duration of 17.6 (9.9–26.2 years) years [19]. However, disease duration is not always consistently accounted for, and most studies on DME prevalence are cross-sectional, limiting our ability to fully understand the natural history of DME.

Our study demonstrates a marked increase in DME prevalence with prolonged T1D duration, particularly after 20 years. Our findings corroborate the results of earlier studies, such as WESDR, which reported a 25-year incidence of DME of 29%, and the Diabetes Control and Complications Trial/Epidemiology of Diabetes Interventions and Complications (DCCT/EDIC), which reported a 30-year incidence of CSME of 37% [5, 20]. However, our study extends these observations by analyzing an even longer period of up to 65 years, and the increasing risk with longer disease duration is undeniable: the cumulative risk of DME escalates from 7.6% at 20 years to 51.1% at 65 years of T1D duration. When we divided our cohort into patients born before and after the year 2000, an initial observation suggested that those more recently diagnosed were less likely to develop DME. This conclusion could be plausible, given that patients diagnosed more recently likely had access to better therapeutic options for a greater proportion of their disease duration. However, after performing a multivariate analysis adjusted for disease duration, this difference was no longer observed. This outcome suggests that, although improved glycemic control may be achievable, the effect is offset by the duration of the disease. This significant increase underscores the critical need for intensified surveillance and intervention strategies beyond the 20-year mark, a period identified as a potential temporal cut-off point for increased DME risk. In an era of increased survival of patients with T1D, these findings may significantly influence clinical decision-making, supporting the need for early and continuous monitoring in patients with long-standing T1D. A study assessing the quality of life in patients with diabetes demonstrated that greater declines in visual acuity were associated with more significant reductions in quality of life [21]. Another study reported that patients with T2D with DME experienced a lower quality of life compared to patients with T1D with only DR, as well as patients with glaucoma or cataracts [22]. Although these studies included patients with both T1D and T2D or only patients with T2D, it can be inferred that, given the younger age and higher employment likelihood among those with T1D, this population may experience a more significant impact from DME.

This study also highlights a strong association between the severity of DR and the incidence of DME, with more than half of the patients with DME presenting with either severe NPDR or PDR. This pattern differs from what is commonly observed in T2D. Romero-Aroca et al. reported a higher prevalence of DME in T2D than in T1D [23, 24], although findings across studies remain somewhat inconsistent. Additionally, T1D patients tend to present with more severe retinopathy [23], which supports our observation that, in T1D, DME development is closely linked to retinopathy severity. This relationship cannot be overlooked, as disease duration is a key factor in the progression of DR. To account for this potential confounder, our study performed an analysis adjusted for disease duration. These findings support the concept of a progressive DR course leading to DME, reinforcing the importance of regular macular assessment as DR severity advances, particularly in light of the increased life expectancy observed in patients with T1D. Early treatment of DME has been associated with better visual outcomes compared to observation alone [11, 25], underscoring the importance of timely diagnosis, particularly in T1D patients who are typically younger and within their working-age years.

It is also important to emphasize the association between the presence of DME and other microvascular complications, namely nephropathy and neuropathy. These findings are consistent with previous studies, such as the one by Romero-Aroca et al., which reported that, over a 10-year follow-up of a T1D cohort, 11.07% of patients developed DME, and among those, 62% had nephropathy (*p* < 0.001) [26]. The higher prevalence of nephropathy observed in that study may be explained by the fact that most patients were not undergoing intensive insulin therapy. Regarding neuropathy, a study by Klein et al. found that patients with sensory neuropathy had more than threefold increased odds of having prevalent DME [27]. As for macrovascular complications, our study did not identify any significant association. This finding is consistent with the results of a study by Eriksson et al. conducted in a Finnish population, where the presence of CSME was not associated with the risk of stroke after adjusting for diabetic kidney disease [28].

Age at T1D diagnosis was found to be an independent risk factor to develop DME. Interestingly, this supports the previous research by Hietala et al., suggesting that older patients are more susceptible to microvascular complications, including DME, possibly due to a diminished ability to respond to hyperglycemia [17]. A previous study by Klein et al. demonstrated that diabetic children exhibited a lower susceptibility to developing retinopathy prior to puberty, irrespective of disease duration. However, this protective effect diminished following the onset of puberty [29]. This increased vulnerability might result from a reduced capacity to maintain the integrity of the retinal endothelial barrier, leading to a higher likelihood of DME development. Additionally, the association between older age at diagnosis and increased DME risk may be attributed to a delayed diagnosis of T1D, resulting in a prolonged period of untreated hyperglycemia and increased cumulative glycemic burden. This observation highlights the importance of considering age at diagnosis in risk assessments and suggests that older patients at diagnosis might require more intensive monitoring for DME. Further research is needed to explore the underlying mechanisms and to establish a clearer understanding of how age-related factors influence DME susceptibility in T1D patients.

Although elevated HbA1c levels did not remain statistically significant in the multivariate analysis, it was previously identified as a single significant predictor of DME. The WESDR and DCCT/EDIC established a strong link between chronic hyperglycemia and the risk of developing microvascular complications, such as DME, and considered it as an independent variable [5, 20]. This difference may be explained by our retrospective way of collecting data to establish the mean HbA1c, which is a limitation of our study. However, the results underscore the necessity of maintaining optimal glycemic control to reduce the likelihood of DR supporting current clinical guidelines that advocate for tight glucose management in T1D patients. Future studies incorporating continuous glucose monitoring will be crucial to provide a more dynamic, accurate, real-time assessment of glucose fluctuations, which may better predict the relation between glycemic control and DME risk.

Despite not finding a statistically significant correlation between BMI and DME, the role of obesity in the context of metabolic syndrome and its potential impact on microvascular complications remains a critical consideration. The research by Klein et al. demonstrated that higher BMI is associated with an increased risk of DR, and by extension, may contribute to DME. Their findings indicated that for each 4 kg/m^2^ increase in BMI, the risk of developing DR increased by 16% and the risk of progressing to PDR increased by 21% [30]. While the current study did not observe a direct link, the potential influence of BMI on DME warrants further investigation. Future studies could provide a more detailed analysis of the relationship between BMI and DME, considering additional factors such as body fat distribution and metabolic health.

Even though this study found that SBP was significantly higher in patients with DME, suggesting a potential role of systolic hypertension in the pathogenesis of DME, in the multivariate analysis, SBP did not show a significant association with DME. DBP was not significantly associated with DME in the univariate analysis (*p* = 0.349). The research by Adler et al. identified blood pressure control as a critical factor in preventing microvascular complications in diabetes, including DR and DME [31], although this study was conducted on patients with T2D. Differences between both studies could be explained by the fact that the physiopathology of DME in T1D and T2D are different.

### Strengths and limitations

The major limitation of this study is its retrospective design. Data was collected from existing medical records, which were not originally intended for research purposes, potentially leading to incomplete or inaccurate information. Additionally, the retrospective nature of the study may introduce selection bias, as only patients who were actively followed within the healthcare system and had available medical records were included. This selection bias could result in an underestimation or overestimation of the prevalence of DME, particularly among patients who were lost to follow-up or who sought care in other healthcare settings. Furthermore, the study’s reliance on data from a single tertiary care center limits the generalizability of the results. Another notable limitation pertains to the method by which key clinical variables such as HbA1c, BMI, SBP, and DBP were calculated. These variables were derived as mean values from the records available in the electronic medical system. However, given the potential fluctuations in these measures over time, this approach may not fully capture their variability, which could be significant in understanding the progression of both T1D and DME. A major strength of our study lies in its representative sample, which reflects the prevalence of T1D in the Northern Region of Portugal, enhancing the confidence in our findings. Moreover, as a longitudinal study with a wide follow-up period extending up to 65 years, it provides valuable insights into the natural history of DME in patients with T1D. Additionally, the evaluation of risk factors for DME development further enriches the study, making it a comprehensive contribution to the understanding of this condition.

## Conclusion

This study underscores the significant time-dependent nature of DME development in individuals with T1D, particularly highlighting an exponential increase in prevalence after 20 years of disease duration. These findings reinforce the critical need for long-term, vigilant monitoring of patients with T1D, emphasizing the importance of early detection and proactive management strategies to mitigate the progression of DME.

## Data Availability

The data supporting this study cannot be shared publicly due to ethical and legal restrictions protecting patient confidentiality and privacy.
